# Reducing Pending Glycated Hemoglobin (HbA1c) Patient Reports in a Tertiary Care Centre in North India: A Quality Improvement Project

**DOI:** 10.7759/cureus.101705

**Published:** 2026-01-16

**Authors:** Koushik Biswas, Altaf Ahmad Mir, Vivek Kushwaha

**Affiliations:** 1 Biochemistry, All India Institute of Medical Sciences, Raebareli, IND; 2 Biochemistry, Maharshi Devraha Baba Medical College, Deoria, IND

**Keywords:** clinical laboratory quality management, fish bone analysis, glycated hemoglobin (hba1c), hospital information system (his), laboratory information system (lis), laboratory quality improvement, pdsa cycle, quality improvement, root-cause-analysis, run chart

## Abstract

Introduction

In early August 2023, several outpatients at All India Institute of Medical Sciences (AIIMS), Raebareli, India, complained that their HbA1c reports were not ready as promised. Some required to give a repeat sample, which caused patient dissatisfaction and increased laboratory workload. The aim of this quality improvement (QI) project was to reduce pending glycated haemoglobin (HbA1c) reports by improving laboratory and sample collection workflows within a 12-week period.

Methods

A team was formed to fix the problem. Process mapping, fishbone analysis and two successive Plan-Do-Study-Act (PDSA) cycles were undertaken over 12 weeks. The first PDSA cycle was in the laboratory, and the second was in the sample collection area. Data was extracted in Microsoft Excel spreadsheet. Data was analysed in Microsoft Excel and GraphPad. Fisher's Exact Test was used to determine if the improvement from this QI project was statistically significant.

Results

Process mapping and fishbone analysis revealed that the barcode scanner of the HbA1c analyser was non-functional, hence the technician manually entered the results in a laboratory register and afterwards entered those in the Hospital Information Management System (HIMS). In PDSA cycle 1, the team noticed that the manual register contained only laboratory ID (unique laboratory identification number) and HbA1c values. The team instructed the laboratory technician to include the patient’s registration number in the manual register, besides the laboratory ID and test value. In PDSA cycle 2, the team observed that after raising a test request, a few patients had departed the collection area without giving a sample. The team advised the nursing officers in the sample collection centre to prepare a list of patients (with their hospital registration number) who had raised the HbA1c test requests, but departed without giving a blood sample, and send this list daily to the laboratory technician after sample collection hours. These interventions reduced the pending HbA1c reports from 1.69% to 0.19% over 12 weeks (p=0.016).

Conclusion

Reduction in pending reports decreased the number of patient complaints and improved the laboratory workflow process. Continuous assessment and documentation of pending work, clarity on tasks needed, and regular staff meetings were essential for this success.

## Introduction

Problem 

In the first week of August 2023, many patients visited the Biochemistry laboratory of our institute and complained that they were not receiving their glycated haemoglobin (HbA1c) reports as promised from the report dispatch counter. This was inconvenient for the patients as it delayed their consultation. Some patients had to provide a repeat blood sample, which led to inconvenience and dissatisfaction. These patients risked a delay in diagnosis and treatment.

All India Institute of Medical Sciences (AIIMS), Raebareli, is a government-run hospital located in the state of India. The patients (except those under health schemes) pay for their diagnostic investigations. The cost of the investigations is kept at par with similar government institutes to make it affordable for patients belonging to all socioeconomic classes. The institute provides diagnostic services in the field of biochemistry, pathology, microbiology, transfusion medicine and radiology. During the study period, the routine work of the Biochemistry laboratory was carried out by two consultants, one resident, a few laboratory technicians and laboratory attendants. The Biochemistry laboratory is equipped with a fully automated clinical chemistry analyser, an immunoassay analyser, an HbA1c analyzer and an ELISA reader. The institute has a Hospital Information Management System (HIMS) to track patient treatment. The HIMS also reflects laboratory work processes and the status of patient reports. In the first week of August 2023, the HIMS reflected that 1.69 % of HbA1c reports were pending.

Background

A common criterion for evaluating laboratory performance is timeliness. Clinicians rely on timely results to attain early diagnosis and care for their patients. One of the most crucial instruments used by clinicians to effectively oversee the standard and security of patient treatment is timely laboratory reports [1]. Timely validation of laboratory findings aids clinicians in promptly diagnosing patients, which results in a successful treatment strategy [2]. Studies indicate that longer turnaround time increases rates of morbidity and mortality [3]. Research conducted at teaching hospitals and tertiary institutions has revealed a number of interventions during the pre-analytical, analytical, and post-analytical stages that might enhance timeliness. Adopting optimal phlebotomy procedures, bar coding samples, implementing a laboratory information system (LIS), utilizing fully automated equipment, training technical personnel, and numerous other actions that could aid in delay reduction were recommended by this research [4-6].

Fixing workflow subprocesses improved the compliance of timely report generation, according to another study on the timeliness of surgical pathology reports. The study's main objectives were to create log sheets that pathologists could attach to their requests, remind pathologists every day to confirm finished reports on the same day, and resolve login issues with the impacted system [7]. Turnaround time (TAT) is the most widely used metric for timeliness. A review of the literature identifies a number of distinct methods for defining TAT. TAT can be categorized by test analyte (e.g., potassium), priority of patient (e.g., urgent or routine), patient population served (e.g., inpatient, outpatient, emergency department), and activities included (e.g., from the time of ordering or from the time of sample reception in the laboratory). Lundberg listed nine processes for conducting a laboratory test: ordering, collection, identification, transportation, preparation, analysis, reporting, interpretation, and action [8].

Rationale

Many laboratories limit their definition of TAT to intra-laboratory operations, despite the fact that the laboratory can and possibly should be involved in all the processes [8]. According to some researchers, laboratory TAT is the "time from receipt of the specimen in the laboratory till the availability of the result", whereas total TAT is defined as "time from the physician's request to the time the physician views the result"[9]. Existing literature highlights that a single definition of TAT is insufficient for all test kinds and contexts. While some suggest that the TAT definition should be based on the priority of the sample (routine or urgent/STAT) and type of patient served (outpatient department/non-emergency inpatient department/emergency/casualty service). They came to the conclusion that in order to use TAT as a quality metric for laboratory services, hospitals must develop their own TAT in conjunction with doctors, laboratory staff and clients [10].

In our laboratory, we consider TAT as the time from the collection of the patient’s sample to the report generation in the online system. During the study period, the timing for sample collection of patients attending the outpatient clinics in our institute was 8:00 am to 3:00 pm. The reports of patients attending the outpatient clinic had to be made available by 8:00 am on the next day, as the patient might come for a follow-up visit. This study was needed as many patients complained that they were not receiving their glycated haemoglobin (HbA1c) reports when they came to the hospital to collect them.

Aim

The aim of this quality improvement project was to reduce pending glycated haemoglobin (HbA1c) reports by improving laboratory and sample collection workflows within a 12-week period.

## Materials and methods

Study setting

This prospective quasi-experimental (pre- and post-intervention) quality improvement study was conducted at the Biochemistry Laboratory and Sample Collection Centre of All India Institute of Medical Sciences (AIIMS), Raebareli, Uttar Pradesh, India. AIIMS Raebareli is a tertiary care teaching institution with around 600-bed admission facility in the Inpatient department (IPD). About 2500 patients attend the Outpatient Department (OPD) of the institute daily for consultation. The study was conducted between August 2023 and October 2023 over a period of 12 weeks. The SQUIRE 2.0 Checklist was used to report the quality improvement (QI) work [11].

Intervention

The QI team, comprising two consultants and a resident doctor from the Biochemistry laboratory, was formed. The personnel identified in the process were categorised into groups, namely, (a) consultants and residents validating Biochemistry laboratory reports, (b) laboratory technicians, (c) laboratory attendants, (d) nursing staff, and (e) supporting staff in the sample collection centre. The QI team first informed all the groups about the problem that needed to be addressed. The nursing staff in the sample collection centre were responsible for the patient’s sample collection. The supporting staff present there helped with queue management. The laboratory attendants transported the patient samples to the laboratory and centrifuged them. The laboratory technicians ran the patient samples in the different analysers, such as the HbA1c analyser. The consultants and the resident doctor validated the patient report. The QI team observed all groups every day during their working hours. Adequate care was taken not to disturb the work routine and workflow. It took around 15-20 minutes to observe a group. The observations were done openly, with the groups knowing that the QI team may observe them anytime during the study. The identities of group members being observed were kept confidential.

Measurement and analysis

We applied a Model for Improvement (MFI) approach in this QI project [12]. This comprised of two parts, strategy and action. To facilitate this, we conducted a fishbone analysis. This was a structured brainstorming of the possible contributing issues. This prompted our staff to think about the various types of underlying problems (pre-analytical, analytical and post-analytical), including those pertaining to human errors, processes or equipment, and the environment.

Thereafter, our action plan consisted of Plan, Do, Study, Action (PDSA) cycles. In the “P” stage, we planned for an intervention and data collection method. In the “D” stage, we implemented the intervention. We analysed our data in the “S” stage and compared our results to our pre-intervention predictions. This helped us determine what actions needed to be taken in the “A” stage. We completed two PDSA cycles. The first PDSA cycle was in the Biochemistry laboratory, and the second was in the sample collection centre.

The percentage of pending HbA1c reports was calculated from the laboratory workflow in the Hospital Information Management System (HIMS) of AIIMS Raebareli. The calculation was done by the first author at three intervals, viz., at the beginning of the study (first week of August 2023), at six weeks after the first PDSA cycle (mid-September 2023), and at 12 weeks after the second PDSA cycle (mid-October 2023).

Data was extracted from the Hospital Information Management System (HIMS) and entered into a Microsoft Excel spreadsheet (Microsoft Corp., Redmond, WA, USA). The data entry was done by the first author, and the accuracy was cross-checked by the third author. Thereafter, we carried out data analysis in Microsoft Excel and GraphPad (https://www.graphpad.com/; GraphPad Software, San Diego, CA, USA) programs. The categorical data of pending and completed HbA1c reports in the pre-intervention and post-intervention periods were presented as frequency and percentage. Fisher's Exact Test was used to determine if the improvement from this QI project was statistically significant, as one cell count was less than five. A p-value of less than 0.05 was considered to be statistically significant.

Ethical consideration

This project was deemed to be an improvement project and not a study on human subjects. The work focuses on reducing pending work in the laboratory and not on patient data. Hence, ethical approval was not required.

## Results

Phase 1 (Pre-intervention phase) (early to mid-August 2023)

**Figure 1:**
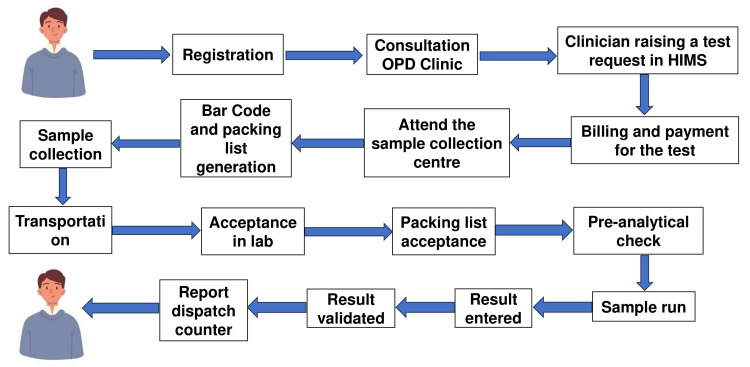
Patient journey mapping OPD: Outpatient Department; HIMS: Hospital Information Management System

We attempted to map a patient’s journey attending our hospital outpatient department (OPD) (Figure 1). This includes registration, consultation in the OPD clinic, billing for the tests, visiting the sample collection centre for phlebotomy and collecting the report on the next working day from the report dispatch counter.

**Figure 2:**
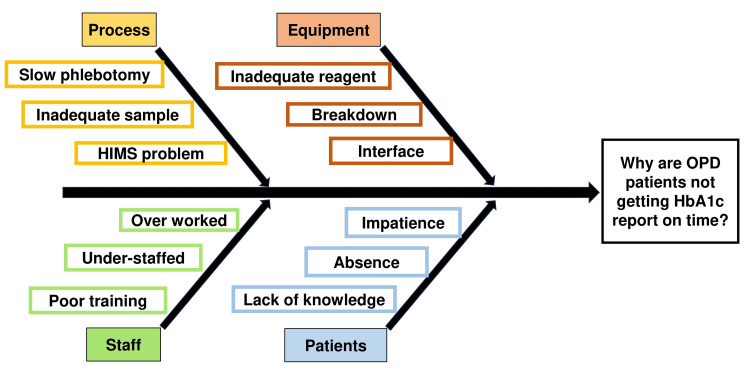
Fishbone diagram showing the root cause analysis HIMS: Hospital Information Management System; OPD: Outpatient Department; HbA1c: Glycated Hemoglobin

Next, we conducted meetings with our staff both from the Biochemistry laboratory and in the sample collection centre. We asked our staff for their opinion on the difficulties they faced in completing their work and the difficulties faced by the patients who arrived to give a sample. We also sought the staff’s suggestions. These data were interesting and helped encourage participation and develop ideas for the QI project. The root cause analysis was done with the help of the Fishbone diagram (Figure 2).

Phase 2 (first PDSA cycle) (mid-August to mid-September 2023)

The HIMS revealed that 1.69 % of HbA1c reports were pending at the beginning of the study. The first intervention was undertaken in the Biochemistry laboratory. In our laboratory, the analysers are interfaced with the HIMS software. Normally, the barcode label of the sample was detected by the analyser via this interface. After the sample run, the interface software transfers the test result from the analyser to the HIMS. Our team observed that the interface of the HbA1c analyser was not working. As a result, the technician was manually entering the HbA1c test result in a register. The technician used to enter the laboratory ID number and test result in the register without entering the registration number or the name of the patient. This posed a challenge during the manual entry of the test results in the HIMS. The HIMS system reflected the patients' (name, registration number and laboratory ID) with pending HbA1c test results, but it was challenging to track them from the manual register. In PDSA cycle 1, the technician was instructed to include the patient’s registration number in the manual register, besides their laboratory ID and test value. This led to a definite improvement, with the pending HbA1c reports reducing from 1.69 % in the pre-intervention stage to 1.20 % at the end of the first PDSA cycle.

Phase 3 (second PDSA cycle) (mid-September to mid-October 2023)

**Figure 3:**
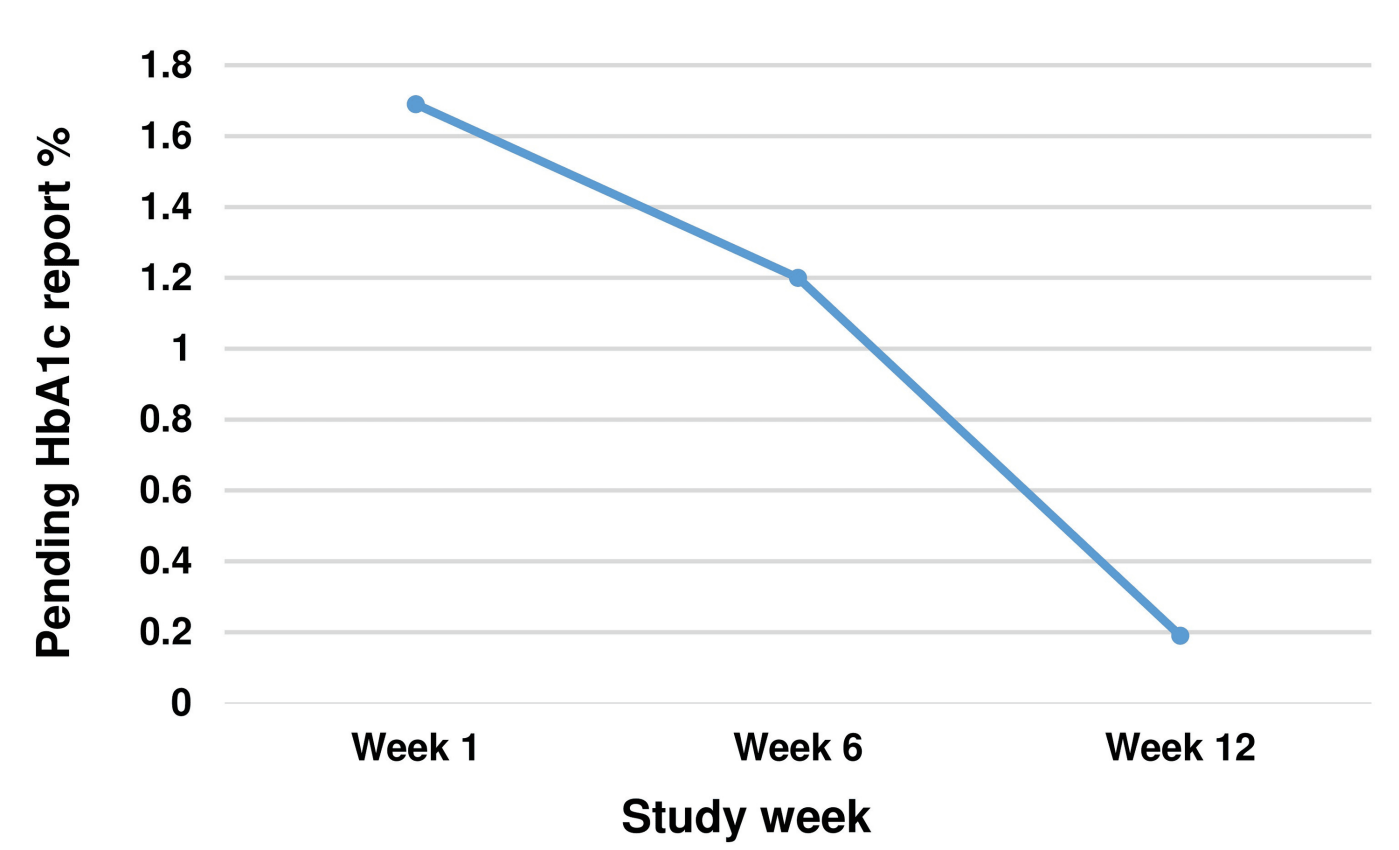
Run chart showing percentage of pending HbA1c reports over the study weeks HbA1c: Glycated Hemoglobin

The second intervention was undertaken in the sample collection centre. The patients arriving for phlebotomy submitted their billing receipt at the counter of the sample collection centre and thereafter sat in the waiting area of the centre till they were called as per their serial number. Our team discussed with the nursing officers and other staff posted in the sample collection centre. We came to know that a few patients, instead of waiting, left the sample collection centre for other work or radiology investigations, and did not return. Their test request was raised in the system, bar barcode was generated, but they did not appear when they were called for phlebotomy. Hence, the HIMS reflected their name in the pending HbA1c work list. Our team suggested that the nursing officer of the sample collection centre prepare a list of such patients (name and registration number) who left the centre without giving a blood sample and convey this list to the laboratory technician in the Biochemistry laboratory every day after the collection time was over (after 3 pm). This helped the technician mention in the HIMS system that they had not given a sample, hence, their report was pending. This led to a significant improvement, with the pending HbA1c reports coming down to 0.19 %. A run chart is an effective tool for assessing the effect of QI projects. The data in the run chart, shown in Figure 3, highlights that this QI project helped to achieve its target to reduce the pending HbA1c worklist.

In the pre-intervention period, we observed that 11 (1.69 %) HbA1c reports were pending, while 637 (98.31 %) were completed. In the post-intervention period, we observed that one (0.19 %) HbA1c report was pending, while 524 (99.81 %) were completed. On statistical analysis, we observed a statistically significant decrease in the pending HbA1c reports (p = 0.016) (Table 1).

**Table 1:** Comparison of the HbA1c worklist between the pre-intervention and post-intervention period. HbA1c: Glycated Hemoglobin

Timeline	HbA1c reports pending	HbA1c reports completed	Fisher's exact test p-value
Pre-intervention	11 (1.69 %)	637 (98.31 %)	0.016
Post-intervention	1 (0.19 %)	524 (99.81 %)	

## Discussion

Turnaround time (TAT) is a major parameter reflecting a laboratory’s service, and many clinicians consider it to judge the quality of the laboratory. Unsatisfactory TAT leads to many complaints from the patients and the clinicians. It also takes a lot of time and effort from the laboratory employees to resolve concerns about pending laboratory reports. Many laboratories have struggled to improve their TATs despite advancements in automation, transportation systems, and analytical technologies [13]. This quality improvement project in a resource-constrained setting taught us a number of lessons. We discovered that minor changes can result in significant patient satisfaction. Interfacing the analyser with the HIMS helps to reduce the time and manpower needed to enter the test results into the HIMS [14]. In this study, we observed that the interface of the HbA1c analyser was not working. This required manual entry of reports, which needed more time and led to a greater number of pending reports. Interfacing can be unidirectional or bidirectional. A unidirectional interface transports data from the analyser to the Laboratory Information System (LIS) and is suitable for small laboratories. A bidirectional interface transports data from the analyser to the Laboratory Information System (LIS) and vice versa [15]. In our laboratory, the barcode label of the sample could not be read by the HbA1c analyser, resulting in the interface not working. The matter was discussed with the company manufacturing the barcode for our laboratory, which did further root cause analysis and helped us to resolve the issue in the long run.

Next, we observed that involving individuals and enhancing cooperation and communication skills among coworkers made it easier to implement the improvement ideas and assist in identifying potential issues and roadblocks that might arise during the improvement journey [7]. In our case, communication between the sample collection centre staff and the laboratory technicians helped us to know the patients who had departed without giving a blood sample. A queue management system in the hospital can help to reduce patient waiting time and improve patient satisfaction. Under this system, the patients are provided with a paper token or a mobile message mentioning their serial number in the sample collection centre. A display reflects the serial number of the patient currently undergoing phlebotomy in different workstations. This helps the patients in the waiting area know their expected waiting time and reduces their anxiety [16].

The strength of our study is that we addressed a real-world problem with direct patient impact. The interventions undertaken were simple, low-cost, and practical. Staff engagement across departments was a major strength and helped to identify and develop effective solutions. As a consequence of this improvement project, a repeat phlebotomy of the concerned patients was not required. A root-cause analysis helped us to identify the interface problems of the HbA1c analyzer, and collaboration with the interface software company helped us resolve this issue in the long run. This eased the work of our laboratory staff. Limitations included that there was no patient representative or a clinician from the outpatient clinic in this project. As a result, patient or clinician feedback was not available. However, patients' complaints about pending HbA1c reports reduced significantly, which indirectly reflected increased client satisfaction. This was a process improvement study and not a causal clinical trial. The study results reflect an association and not proof of causality. The study is limited to a single centre, which limits generalizability. A follow-up check in HIMS three months after the project completion helped us to confirm that the improvement from this QI project was long-lasting.

## Conclusions

Our team successfully reduced pending HbA1c reports over 12 weeks. Reduction in pending laboratory reports increased patient satisfaction. Continuous assessment and documentation of pending work, clarity on tasks needed, regular staff meetings and staff communication were essential for this success. Periodic measurement of delays, fishbone analysis for possible reasons of delays, root cause analysis and using PDSA cycles were essential for this success. Our laboratory staff had been inspired to increase their effort in order to minimise test report delays by the project's consistent improvements.
